# SETD3 Methyltransferase Regulates PLK1 Expression to Promote *In Situ* Hepatic Carcinogenesis

**DOI:** 10.3389/fonc.2022.882202

**Published:** 2022-07-14

**Authors:** Meng Cheng, Qingmiao Yang, Yafei Liu, Meng-Jie Zhao, Xinyuan Du, Jiaqi Sun, Wen-Jie Shu, Zan Huang, Jianping Bi, Ximing Xu, Hai-Ning Du

**Affiliations:** ^1^ Hubei Key Laboratory of Cell Homeostasis, RNA Institute, College of Life Sciences, Renmin Hospital of Wuhan University, Wuhan University, Wuhan, China; ^2^ Cardiovascular Center, Beijing Tongren Hospital, Capital Medical University, Beijing, China; ^3^ Department of Radiation Oncology, Hubei Cancer Hospital, Tongji Medical College, Huazhong University of Science and Technology, Wuhan, China; ^4^ Department of Oncology, Renmin Hospital of Wuhan University, Wuhan, China

**Keywords:** hepatocellular carcinoma, SETD3, PLK1, gene expression, *Sleep Beauty* system, inhibitor

## Abstract

**Background:**

The development of a new strategy to overcome chemoresistance to hepatocellular carcinoma (HCC) treatment is a long-standing issue. We have previously found that upregulated SETD3 levels are closely correlated with HCC. This study aims to explore the mechanism underlying how upregulation of SETD3 promotes liver carcinogenesis.

**Methods:**

RNA-Sequencing analysis was used to explore the correlation of SETD3 with regulatory targets. *In vitro* assays including cell proliferation and migration were performed to study the oncogenic roles of SETD3 and PLK1. Western blotting, immunohistochemical staining, and blood biochemical assays were performed to examine protein expression or pathological index in tumor tissues and mice liver tissues. Luciferase reporter system and chromatin immunoprecipitation assays were used to explore the mechanism.

**Results:**

We revealed that SETD3 regulates gene expression in subgroups, including cell division, cell proliferation, and cell cycle, in hepatocellular tumor cells. We found that SETD3 upregulation is associated with elevated PLK1 level in both hepatic tumor cells and clinical liver tissues. We further showed that overexpression of SETD3 promoted tumor cell proliferation and migration, whereas inhibition of PLK1 activity attenuated these phenotypes caused by SETD3. By taking advantage of the *Sleep Beauty* transposase system, we confirmed that upregulated mouse Setd3 promoted hepatic carcinogenesis *in situ*, but knockdown of mouse *Plk1* mitigated Setd3-promoted tumorigenesis in mice. Mechanistically, we showed that SETD3 could be recruited to the promoter of *PLK1* gene to facilitate *PLK1* transcription.

**Conclusions:**

Our data demonstrate that elevated SETD3 may promote HCC by enhancing PLK1 expression, which suggests that SETD3 may act as a potential drug target combined with PLK1 inhibition to treat HCC.

## Introduction

Hepatocellular carcinoma (HCC) ranks as the fourth deadliest cancer, with the sixth most common incidence in the world (1). It is estimated that more than 1 million individuals will be affected by liver cancer annually by 2030 (2). During the past decades, although growing studies on the epidemiology and molecular and genetic factors have made great progress to the strategies for prevention, surveillance, early diagnosis, and treatment of HCC, a significant population of HCC patients still present at an advanced stage in many parts of the world (3). Hence, comprehensive understanding of the unknown factors and deciphering the underlying molecular mechanisms are still necessary.

Recently, epigenetic dysregulation has been demonstrated to play a crucial role in the initiation, progression, and metastasis of HCC by altering gene expression through DNA methylation, histone modification, chromatin remodeling, or changes in levels of different types of RNAs (4, 5). For example, changes in levels of DNMT1 and DNMT3, two DNA methyltransferases, contribute to alter DNA methylation levels of certain genes (such as c-Met and MTA1), which is closely associated with metastasis of HCC (6). In addition, histone methylation is also linked with metastasis and proliferation of HCC. It has been reported that upregulation of the methyltransferases EZH2, SETDB1, and G9a are tightly associated with aggressive clinicopathological features of HCC (6). Interestingly, histone demethylases KDM5C and JARID1B are found to be abundantly expressed in invasive human HCC cells and are correlated with distant metastasis of HCC (6). Based on the reported studies *in vivo* and *in vitro*, in which epigenetic modifiers effectively involve in HCC metastasis and proliferation, these epigenetic modifiers could be appreciated as important therapeutic targets for HCC besides chemotherapy or targeted therapies (7). Actually, histone deacetylase inhibitors (HDACi) have been implicated as a therapeutic option in liver cancer (7). Moreover, emerging evidence suggest that many epigenetic regulators could be utilized as potential biomarkers in liquid biopsy for the diagnosis and prognosis of HCC (8). Thus, an extensive understanding of the epigenetic modifications associated with HCC could provide the basis for developing advanced approaches to treat this disease.

Very recently, SETD3, a putative methyltransferase, has been discovered as the first identified histidine N3-methyltransferase in metazoans, which attracts much more attention in the study of the biological roles of SETD3 and protein histidine methylation (9, 10). The biochemical property of SETD3 was initially characterized as a histone H3 Lys4 or Lys36 methyltransferase, which regulates the transcription level of *MyoD*, a key transcription factor, to control muscle cell differentiation (11, 12). In contrast, other results indicate that SETD3 is incapable of methylating histones *in vitro*, and silencing endogenous SETD3 gene fails to alter histone methylation levels (9, 13). Regardless of such paradox, several studies suggest that SETD3 directly regulates gene expression, thereby involving in multiple physiological and pathological processes. For instance, SETD3 is required for p53 recruitment to activate its target gene expression (14). Dissociation of SETD3 and FoxM1 from *VEGF* gene promoter under hypoxia leads to elevated expression of vascular endothelial growth factor (VEGF), which is required for vascular development (15). Moreover, SETD3 downregulates the expression of kinesin light chain 4 (*KLC4*), which could improve the radiosensitivity of cervical cancer cells (16). In addition, SETD3 regulates the expression of multiple breast-cancer-associated genes, such as *ACTB*, *FBXW7*, *Fascin*, *eNOS*, and *MMP-2*, which suggests it as a promising biomarker for breast cancer prognosis (17). It was also reported that SETD3 promotes CXCR5 expression in CD4^+^ T cells of systemic lupus erythematosus patients (18). All this evidence strongly suggests that SETD3 can regulate the expression of different types of genes that are involved in multiple signaling pathways.

Of note, a line of evidence indicates that SETD3 is closely associated with many diseases, including cancers (19). Our bioinformatic analysis showed that *SETD3* levels are significantly different in 31 tumors compared to normal tissues. In line with this result, a group of studies found that SETD3 are correlated with HCC (13, 20), breast cancer (17), colon cancer (14), B-cell lymphoma (21), cervical cancer (16), renal cell tumors (22), and ovarian carcinoma (23), suggestive of a potential biomarker for prognosis of different cancers. Particularly, our recent study showed that aberrant elevated SETD3 protein levels were detected in HCC tissues, and xenograft mice model confirmed that SETD3 levels are positively correlated with tumorigenesis, which indicates that SETD3 is an important player in liver cancers (13). However, whether and how SETD3 regulates hepatocarcinogenesis *in vivo* is still unclear.

Aberrant cell division is one of the essential events that contributes to tumorigenesis, in which many cell cycle regulatory proteins, including cyclin D/E, PLK1, Aurora A/B, Skp2, and Cdc20, have been frequently detected in various types of human cancers (24). Therefore, how aberrancy in cell cycle progression occurs and how the errors lead to tumorigenesis warrants further investigation. Since our previous study has demonstrated that SETD3 is a cell-cycle-related oncoprotein in liver tumor, we speculated that SETD3 plays an important role in hepatocarcinogenesis.

In this study, we connected the oncogenic role of SETD3 in hepatocarcinogenesis with PLK1 level. By using an *in vivo* transposase system, we confirmed that elevated SETD3 levels are correlated with carcinogenesis in mice liver. Our results demonstrate a critical role of SETD3 in hepatocarcinogenesis and suggests that it may act as a potential drug target combined with PLK1 inhibition to treat HCC.

## Materials and Methods

### Cell Culture

The human liver cell lines, LO2 and BEL7402, were purchased from the Cell Bank of Chinese Academy of Sciences. The HEK 293T cell line was gifted from Dr. Zhiyin Song (the College of Life Sciences, Wuhan University). Cells were cultured in Dulbecco’s modified Eagle’s medium (DMEM) (Cat. SH30243.01, Hyclone, USA) supplemented with 10% fetal bovine serum (FBS, Cat. A0500-3011, Cegrogen, Germany) and 1% penicillin-streptomycin solution (Hyclone). Cells were cultured in a humidified incubator with 5% CO_2_ at 37°C. For cell treatment, various doses of BI6727 (Cat. T6019, Topscience, Shanghai, China) were added into the cell culture medium, and cells were collected at the indicated time points to measure the growth rate by cell proliferation assays (see below).

### RNA Isolation and Reverse Transcription Quantitative PCR

For cell RNA extraction, cells were trypsinized by 0.05% trypsin-EDTA (Cat. S320JV, Shanghai BasalMedia Technologies Co., China) and collected by centrifugation with 500*g* for 3 min. For tissue RNA extraction, ~20 mg of tissue was homogenized and collected with centrifugation. Total RNA was isolated with TRIzol (Cat. 15596018, Thermo Fisher, USA). An aliquot of 1 μg of mRNAs was used for reverse transcription by HiScript II Q RT SuperMix (Cat. R223-01, Vazyme, Nanjing, China), and real-time quantitative PCR (RT-qPCR) was performed according to the manufacturer’s protocol (Cat. MQ10101S, Wuhan Monad Medicine Tech Co., China). The primer sequences used in quantitative PCR experiments are listed in **Supplementary Table S1**.

### RNA Preparation and RNA-Sequencing Analysis

WT or sh*SETD3* HEL7402 cells were collected, and total RNAs were extracted using TRIzol (Cat. 15596026, Invitrogen, USA) and further purified *via* chloroform extraction methods. RNA-Sequencing (RNA-seq) libraries were prepared using KAPA Stranded RNA-Seq Library Preparation Kit according to the manual (Cat. KK8401, KAPA Biosystems, USA), sequenced by Berry Genomics Company (Beijing, China) on Illumina NovaSeq 6000.

FastQC was used for quality control on raw sequence data. The filtered reads were normalized, and differential gene expression analysis was performed using DESeq2 v1.24.0. Gene Ontology was performed by DAVID v6.8. For heatmap, we used R package edgeR to calculate counts per million (CPM) of each gene; then, log (CPM) value was used to make heatmap by using R package ComplexHeatmap. R version 3.6.0 was used for some custom analysis.

### Western Blotting

Western blotting was performed as described previously. Briefly, cells were collected and lysed by radioimmunoprecipitation assay (RIPA) buffer [50 mM Tris–HCl, pH 7.4, 150 mM NaCl, 5 mM EDTA, 1% Triton X-100, 1% sodium deoxycholate, 0.1% sodium dodecyl sulfate (SDS)] with a protease inhibitor cocktail (Cat. B14001, Bimake, China). Tissues were grounded by an electronic homogenizer and lysed by RIPA buffer as well. Protein extracts were separated by SDS polyacrylamide gel electrophoresis (SDS-PAGE) and transferred to polyvinylidene fluoride (PVDF) membranes (Cat. ISEQ00010, Millipore, USA). After blocking with 5% non-fat milk in TBST buffer [10 mM Tris–HCl, 150 mM NaCl, 0.5% (v/v) Tween-20, pH 7.5]. Membranes were probed with the following primary antibodies including mouse anti-SETD3 (1G7, mouse monoclonal, generated by Wuhan Dia-An Company), rabbit anti-PLK1 (Cell Signaling Tech, USA, 4535S), rabbit anti-pT210-PLK1 (Cell Signaling Tech., USA, 9062S), mouse anti-GAPDH (Proteintech, China, 60004-1-Ig), rabbit anti-β-actin (ABclonal, China, AC026), and rabbit anti-Flag (MBL, Japan, PM020). Membranes were washed and incubated with the appropriate secondary antibodies (Jackson ImmunoResearch Laboratory, USA) at room temperature for 1–2 h. Blotting signals were detected by ECL detection reagents (Cat. 36208ES76, Shanghai Yeasen Biotech, China). Prior results were captured by exposure with X-ray films (Fuji Company), and later results were captured by a chemiluminescence imaging system (ChemiScope 3000mini, Clinx Science Instruments Co. Ltd., Shanghai, China). For animal tissues, more than three individual mice samples per group were used for blotting. Densitometric quantification of protein bands was performed using ImageJ (NIH, Bethesda, USA).

### Constructs, Transfection, and Lentiviral Infection

Short hairpin RNA fragments (shRNAs) of mouse *SETD3* containing 5′-GCTGGAGATCAGATTT ACATT-3′ (sh*SETD3*) or mouse *Plk1* containing 5′-GCACCGCAA TCAGGTCATTCA-3′ (sh*Plk1*) were cloned into pLKO.1 vector using the restriction enzymes *EcoR*I and *Age*I (New England Biolabs). Two shRNAs of human SETD3 containing 5′-GCTTTGGTTTGAGAGCAACAA-3′ or 5′-GAAGAAGATGAAGTTCGGTAT-3′ were cloned into plko.1 vector as well. Wild-type (WT) or catalytic inactive Y313A mutant of human SETD3 and WT or catalytic inactive K82M mutant of PLK1 were cloned into pCS2-3xFlag vector. For expression, plasmids were transfected into mammalian cells using lipofectamine 2000 (Cat. 11668019, Invitrogen, USA), according to the manufacture’s protocol. For generation of knockdown cell line, shRNA constructs were transfected into HEK 293T cells along with the helper plasmids pMD2G and psPAX2 to produce virus particles. Knockdown cells were selected by using puromycin and validated by Western blotting.

### Wound Healing Assays

Cells were counted by Automatic Cell Counter (Guangzhou Bodboge Technology Co., Ltd.), and equal numbers of cells were seeded into six-well plates for 24 h, and cell layers were scratched using a 200-μl pipette tip to form wound gaps. Plates were washed twice with pre-warmed phosphate-buffered saline (PBS), and the cells were maintained in DMEM without FBS. The cells were photographed at 0 and 48 h to record the wound width. Three independent replicates were conducted and quantified.

### Transwell Migration Assays

The migratory capability was detected using transwell chambers (8 μm pores, BD Bioscience, Cat. 353097, USA). Briefly, 5,000 cells were plated on the surface of each upper chamber. The media containing 10% FBS was injected into the lower chambers to stimulate cell migration. After 36-h incubation, cells on the lower filter surface were fixed with 4% paraformaldehyde and then stained with 0.1% crystal violet stain solution (Cat. G1063, Solarbio, China). Images were taken by an optical microscope.

### Cell Proliferation Assays

Cell proliferation assays were performed as described previously (13). Briefly, LO2 or BEL7402 cells with expressing SETD3 or PLK1 constructs or stably knockdown of *SETD3* were treated with PLK1 inhibitor BI6727 at the indicated concentrations and measured using a Cell Counting Kit-8 (CCK-8, Cat. EC008, Engreen Biosystem Ltd., New Zealand) according to manufacturer’s instructions. Cell numbers in each well were normalized using Automatic Cell Counter (JSY-SC-031N, Guangzhou Bodboge Technology Co., Ltd.). Each time point was repeated in three wells, and the experiments were independently performed for three times. The effects of cell proliferation were characterized by a growth curve, which was plotted by the value of OD_450_.

### Clinical Specimens

The 18 pairs of liver cancer tissues were collected from patients in Hubei Cancer Hospital. The liver cancer tissue microarrays (HLivH020PG02-M-209, Xi’an Alenabio Company, China), which contained samples from 30 pairs of human hepatocellular carcinoma and adjacent tissues, were purchased from Shanghai Outdo Biotech. Informed consents were obtained from all subjects in accordance with the protocol approved by the individual institutional ethics committees.

### Hydrodynamic Injection and Animal Experiment

WT C57BL/6 mice (8 weeks old) were subjected to hydrodynamic injection procedures as described previously (25). Briefly, 1 μg pCMV-CAT-T7-SB100X, 6.4 μg pT3-Myr-*AKT*-HA, 6.4 μg pT3/Caggs-*NRAS*-V12, and either 6.4 μg pT3-U6-sh*Ctrl* or equal amount of pT3-U6-sh*SETD3* were mixed well and diluted in 2 ml of 0.9% NaCl, filtered and injected into the lateral tail vein of mice within 9 s. Five control mice were injected with 0.9% NaCl solution. After 4 weeks post-injection, all mice were sacrificed in groups to monitor liver tumorigenesis. Livers were separated from each mouse, and liver lesions were diagnosed macroscopically and several biochemical indexes described below.

### Immunohistochemical Staining

Human tissue microarray slides were deparaffinized as described previously (13). For mice tissue staining, mice livers were isolated from the indicated male mice, rinsed in ice-cold PBS, fixed in 4% polyformaldehyde for 48 h at 4°C, and then dehydrated, embedded in paraffin, and sectioned. Tissue sections (4 μm) were used for staining. Slides were then incubated with the α-SETD3 or α-PLK1 antibody at 1:1,000, respectively, followed by biotin-conjugated goat anti-rabbit IgG antibody or goat anti-mouse IgG antibody at 1:10,000, respectively. Detections were performed using a 3,3′-diaminobenzidine (DAB) reagent (Cat. E-BC-K227-S, Elabscience, Wuhan, China), and nuclei were stained with hematoxylin before examination by a microscope. At least four mice per group were used for assay. The intensity score was determined by evaluating staining intensity of positive staining as described previously (13).

### Measurement of Total Cholesterol and Triglyceride

For serum triglyceride (TG) and total cholesterol (TC) content measurements, blood plasma samples were obtained just before necropsy and deposited for 30 min at room temperature. Serum was harvested by centrifugation at 3,000 rpm for 10 min at 4°C. Serum TG and TC contents were determined using the Triglyceride Assay Kit (Cat. A1101-1-1, NJJCBIO, Nanjing, China) and Total Cholesterol Kit (Cat. A1111-1-1, NJJCBIO, Nanjing, China), respectively. The operation was performed according to the manufacturer’s manual.

### Measurement of Alanine Aminotransferase and Aspartate Aminotransferase in Serum

For measurement of the content of alanine aminotransferase (ALT) and aspartate aminotransferase (AST), serum was harvested as described above. The ALT and AST contents were determined using the Alanine Aminotransferase Assay Kit (Cat. C009-2, NJJCBI, Nanjing, China) and Aspartate Aminotransferase Assay Kit (Cat. C010-2, NJJCBIO, Nanjing, China), respectively. The operation was performed according to the manufacturer’s manual.

### Dual-Luciferase Reporter Assays

The luciferase (LUC) reporter plasmid pGL4 vector was generously gifted from Dr. Xiao-Dong Zhang’s laboratory (the College of Life Sciences, Wuhan University). The ~2-kb fragment of *PLK1* promoter extending −1,867 to +27 was amplified by PCR from genomic DNA of HeLa S3 cells as described previously (26) and cloned into pGL4 vector’s multiple cloning site just before *LUC* gene. For Luciferase reporter assays, 60 ng pGL4-*PLK1* (−1,867/+27) reporter vector, 4 ng pRL-TK (Renilla), and 300 ng pCS2-3xHA-SETD3 were transfected into 293T cells. After 24–36 h, cells were lysed, and the luciferase activity was tested according to the manufacturer’s instructions (Cat. E1910, Promega, USA). The primer sequences used to generate various pGL4-*PLK1* reporter constructs are listed in **Supplementary Table S1**.

### Chromatin Immunoprecipitation

Chromatin immunoprecipitation (ChIP) assays were modified as described previously (27). Briefly, ~ 1 × 10^7^ cells were fixed with 1% formaldehyde and then quenched with glycine to a final concentration of 0.125 M. The cells were washed three times with PBS and then harvested in cell lysis buffer (5 mM PIPES, pH 8.0, 85 mM KCl, 0.5% NP-40) with the protease inhibitors and phenylmethylsulfonyl fluoride (PMSF) (1 mM final concentration). DNA in nuclei lysis buffer (50 mM Tris–HCl, pH 8.0, 10 mM EDTA, 1% SDS, and protease inhibitors) was sonicated for 20–25 cycles (high output, 30 s on, 30 s off) using the Bioruptor (Diagenode). After centrifugation, four volumes of ChIP dilution buffer (16.7 mM Tris–HCl pH 8.1, 167 mM NaCl, 0.01% SDS, 1.1% Triton X-100, and 1.2 mM EDTA) were added to the supernatant. The lysate was then incubated with anti-Flag affinity beads (SA042001, SMART LifeSci, China) at 4°C overnight. The beads were washed five times, and DNA was eluted by ChIP elution buffer (0.1 M NaHCO_3_, 1% SDS, and 30 μg/ml proteinase K). The eluent was incubated at 65°C overnight, and DNA was extracted with DNA purification kit (Cat. DP214, Tiangen Biotech, Beijing, China). Purified DNA was analyzed by quantitative PCR.

### Statistical Analysis

Unless otherwise indicated, all data are presented as the mean ± SD from at least three biological replicates, and statistical differences between any two groups were compared by unpaired *t-*tests using Prism 7 software. The relative immunohistochemical staining (IHC) staining scores of human liver tissue microarrays were compared by unpaired Student’s *t-*tests. A *p*-value of <0.05, 0.01, 0.001, or 1 × 10^−4^ was considered statistically significant and marked as “*,” “**,” “***,” and “****,” respectively. “NS” indicates “not significant.”

### Ethics Approval

All procedures were conducted in accordance with the Animal Guidelines for the Care and Use of Laboratory of Wuhan University (No. WDSKY0202001). All mice were housed, fed, and treated in accordance with protocols approved by the Animal Experimentations Ethics Committee of the Wuhan University.

## Results

### Genome-Wide RNA-Seq Analysis Reveals That SETD3 Regulates PLK1 Level in Hepatocellular Tumor Cells

**Figure 1:**
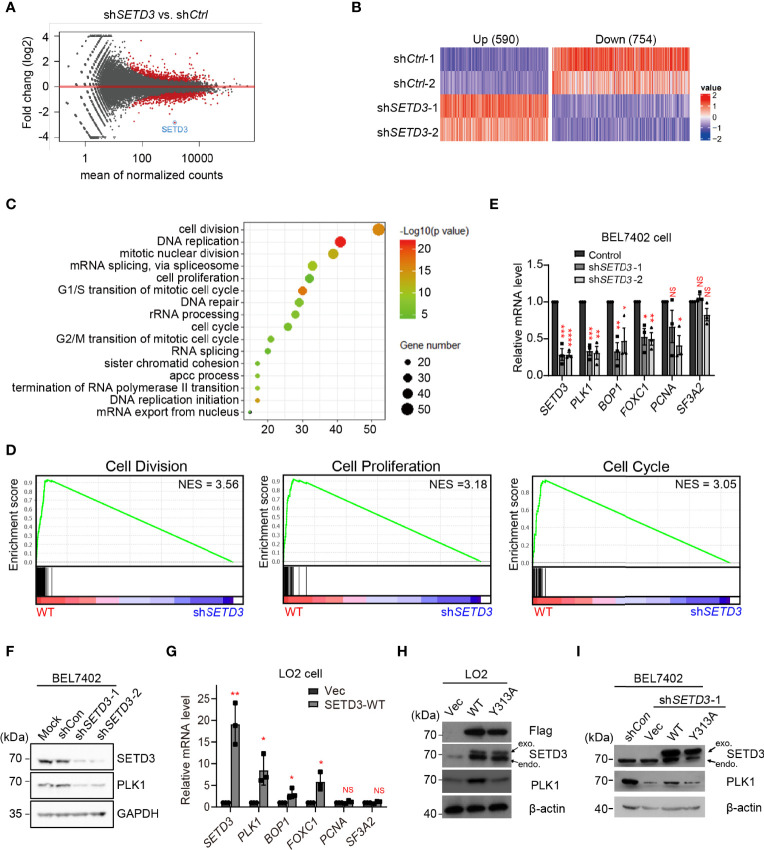
SETD3 regulates PLK1 level in liver tumor cells. **(A)** Volcano plots indicate DEGs from biological duplicates in sh*SETD3* cells relative to sh*Ctrl* cells. The red circles represent differential genes with *p <*0.05. **(B)** Expression heatmap of 590 upregulated genes and 754 downregulated genes from duplicated biological repeats in sh*SETD3* cells compared with sh*Ctrl* cells. The color scales indicate the log2 ratio of relative expression levels (log_2_ >0.5, *p <*0.05). **(C)** GO analyses of biological processes show DEGs in panel **(B)**. **(D)** GSEA shows plots of enrichment in “Cell Division,” “Cell Proliferation,” and “Cell Cycle” pathways in WT and sh*SETD3* cells, respectively. **(E)** RT-qPCR analyses were used to examine mRNA levels of various cell proliferation-related genes upon knockdown of *SETD3* in BEL7402 cells. **(F)** SETD3 and PLK1 protein levels from the indicated samples shown in panel **(E)** were examined using Western blotting. **(G)** RT-qPCR analyses were used to examine mRNA levels of various cell proliferation-related genes upon overexpression of either vector or WT SETD3 in LO2 cells. **(H)** SETD3 and PLK1 protein levels from the indicated samples were examined using Western blotting. **(I)** Rescue experiments were performed in sh*SETD3* cells in transfection with either WT or Y313A mutant of SETD3, respectively, and PLK1 protein levels were examined by Western blotting. exo, exogenous expressed SETD3; endo, endogenous SETD3. Data are presented as mean ± SD from three biological replicates. NS, not significant; *p <0.05; **p <0.01; ***p <0.001.

Our previous study indicated that SETD3 level is upregulated in liver tumor BEL7402 cells, and overexpression of *SETD3* led to mice xenograft tumorigenesis. To investigate underlying mechanism of how SETD3 involves in hepatocellular carcinogenesis *in situ*, RNA-Seq analysis were performed, and the differentially expressed genes (DEGs) in knockdown of *SETD3* cells relative to WT BEL7402 hepatocellular tumor cells were analyzed (**Figure 1A**). We totally identified 1,344 DEGs (threshold set as 1.4-fold change, *p* < 0.05), including 590 upregulated genes and 754 downregulated genes overlapped at two pairs of sh*SETD3* cells vs. sh*Control* cells (**Figure 1B**). Interestingly, gene ontology (GO) analysis indicated that the downregulated DEGs are mainly enriched in cell-cycle-related process, including cell division, cell proliferation, and cell cycle (**Figure 1C**). The gene set enrichment analysis (GSEA) showed that knockdown of *SETD3* is closely correlated with lower expression of genes participated in cell cycle-related pathways (**Figure 1D**). Based on these data, we hypothesized that SETD3 might promote liver tumorigenesis mainly through regulating cell-cycle-related gene expression.

To further verify genome-wide RNA-seq data, expression levels of several top candidates were examined using RT-qPCR analysis. Consistent with whole-genome RNA-seq data, knockdown of *SETD3* significantly reduced mRNA levels of several genes, including *PLK1*, *BOP1*, *FOXC1*, and *PCNA*. In contrast, mRNA level of *SF3A2*, a control gene, was not affected (**Figure 1E**). Consistently, we also observed that knockdown of *SETD3* decreased PLK1 protein levels (**Figure 1F**). Meanwhile, we also chose liver immortalized LO2 cells with overexpression of *SETD3* to phenocopy tumorigenesis of normal liver cell line. Consistently, we found that overexpression of *SETD3* in LO2 cells increased mRNA levels of these genes except the control gene *SF3A2* (**Figure 1G**). Moreover, overexpression of WT but not Y313A mutant of *SETD3* enhanced PLK1 protein levels (**Figure 1H**). We also found that transient expression of WT *SETD3*, but not Y313A mutant, in sh*SETD3* cells was also capable of rescuing protein levels of PLK1 (**Figure 1I**). Altogether, these results indicate that SETD3 regulates PLK1 level in human hepatocellular tumor cells, likely in a catalytic-dependent manner.

### The Effect of SETD3 on Cell Proliferation Is Dependent on PLK1 Kinase Activity

**Figure 2:**
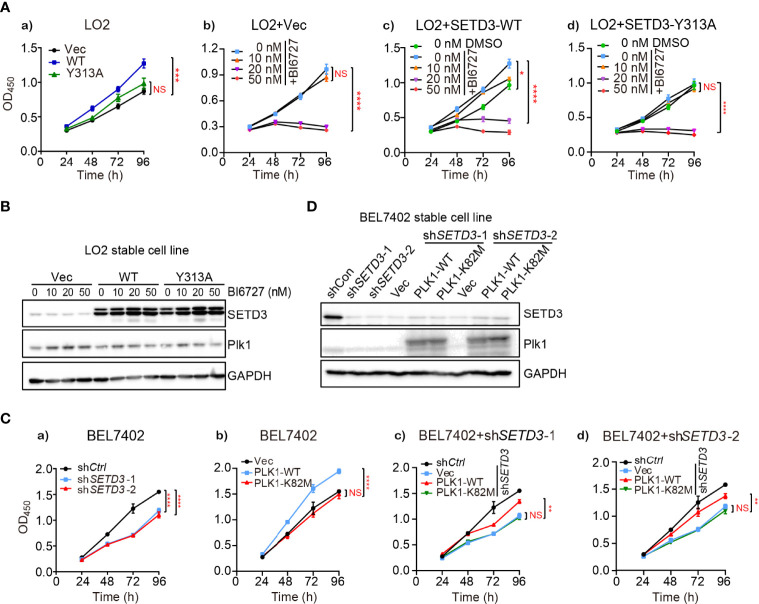
Inhibition of PLK1 activity attenuates the effect of SETD3-enhanced cell proliferation. **(A)** Cell proliferation was determined by CCK-8 assay in LO2 cells transfected with the indicated *SETD3* constructs. Cells were treated with different doses of the PLK1 inhibitor of BI6727 (**A, b–d**). **(B)** Protein levels of SETD3 and PLK1 were examined by Western blotting. **(C)** Cell proliferation was determined by CCK-8 assay in either WT **(a, b)** or sh*SETD3* **(c, d)** BEL7402 cells overexpressing the indicated plasmids including WT or K82M mutant of PLK1. **(D)** Protein levels of SETD3 and PLK1 were examined by Western blotting. Data are presented as mean ± SEM. NS, not significant; *p <0.05; **p <0.01; ***p <0.01; ****p <0.0001.

A growing body of evidence demonstrated that hyperactive PLK1 is required for cell proliferation (28, 29). To investigate whether the effect of SETD3 on liver tumor cell proliferation relies on PLK1 kinase activity, cell growth assays were performed using *SETD3*-overexpressed LO2 cells treated with a PLK1 inhibitor BI6727. We observed that overexpressing WT, but not the Y313A mutant of *SETD3*, facilitates cellular proliferation compared to the control cells (**Figure 2A, a**). As expected, cells treated with higher doses of BI6727 (>20 nM) significantly attenuated cell proliferation, even supplemented with overexpression of WT *SETD3* (**Figure 2A, b–d**). Western blot analysis showed an equal expression of SETD3 and PLK1 in the indicated cells (**Figure 2B**). Furthermore, the effect of PLK1 kinase activity on BEL7402 tumor cell proliferation was also examined. In agreement with previous results, knockdown of *SETD3* retarded cell growth relative to the control cells (**Figure 2C, a**), whereas overexpression of WT, but not catalytic inactive K82M mutant of *PLK1*, promoted cell growth (**Figure 2C, b**). The difference of cell growth rates between WT and K82M mutant does not result from the divergent expression levels, as comparable PLK1 protein levels were displayed (**Figure 2D**). Interestingly, overexpression of WT *PLK1*, but not K82M mutant, can only partially restore decreased cell proliferation caused by knockdown of *SETD3* in BEL7402 cells (**Figure 2C, c,d**), suggesting that PLK1 may only act as one of the downstream targets regulated by SETD3.

### PLK1 Kinase Activity Is Required for Rescuing Attenuated Cell Migration Ability Caused by Downregulation of *SETD3*

**Figure 3:**
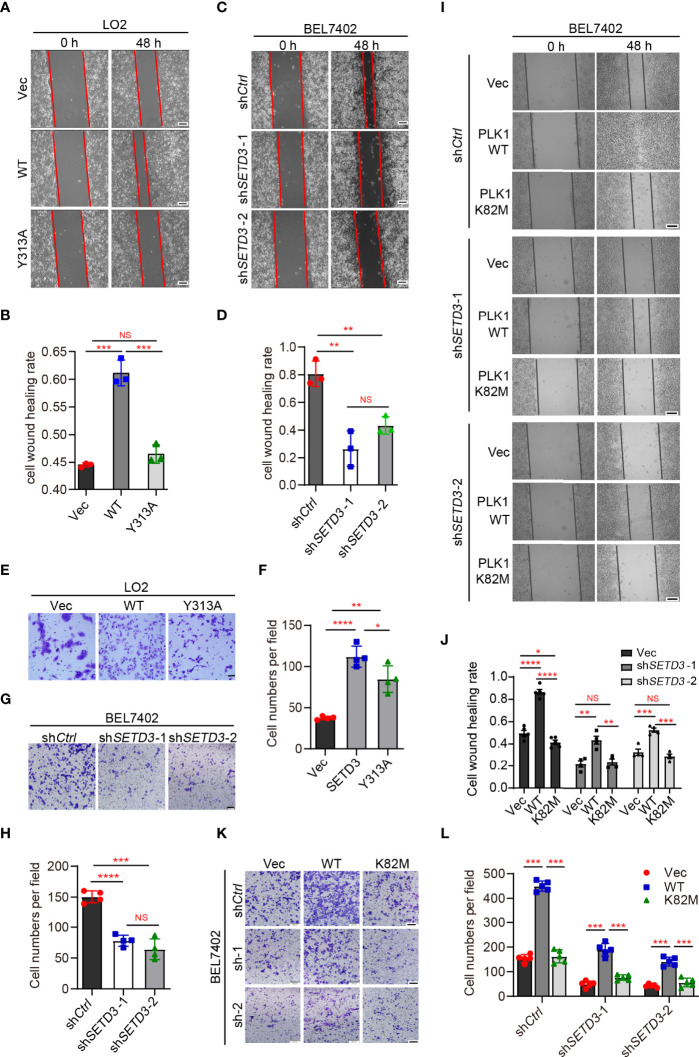
PLK1 kinase activity is required for rescuing decreased cell migration by knockdown of *SETD3*. **(A–D)** Examine cell migration in LO2 cells overexpressed with either WT or Y313A mutant of SETD3 **(A, B)** or in BEL7402 cells transfected with sh*SETD3* construct **(C, D)**. Representative images at the indicated time points were present in panels **(A, C)**, and quantitative data from three independent replicates are shown in panel **(B)** or **(D)**, respectively. *Scale bar*, 500 μm. **(E–H)** Transwell assays were performed in the cells transfected with the indicated constructs. Representative images are present in panels **(E, G)**, and quantitative data from three independent replicates are shown in panel **(F)** or **(H)**, respectively. **(I–L)** Wound healing and cell migration assays were performed in sh*SETD3* BEL7402 cells transfected with WT or K82M mutant of PLK1. Representative images are presented in panel **(I)** or **(K)**, and quantitative data from three independent replicates are shown in panel **(J)** or **(L)**. *Scale bar*, 500 μm. Data are presented as mean ± SD. NS, not significant; *p <0.05; **p <0.01; ***p <0.001; ****p <0.0001.

To explore whether SETD3 is important for cellular migration, wound healing and transwell assays were performed in both LO2 and BEL7402 cells. We noticed that overexpression of WT *SETD3*, but not Y313A mutant, promoted cell migration in normal liver LO2 cells (**Figures 3A, B**). In contrast, knockdown of *SETD3* reduced cell migration in tumor liver BEL7402 cells (**Figures 3C, D**). Consistently, overexpression of WT but not Y313A mutant of *SETD3* facilitated cell migration ability (**Figures 3E, F**), whereas knockdown of *SETD3* reduced cell migration (**Figures 3G, H**). These data suggest the important role of SETD3 in liver cell migration.

Next, we want to know whether the effect of SETD3 on cell migration is dependent on PLK1 kinase activity. Wound healing assays showed that overexpression of WT *PLK1*, but not the K82M catalytic-dead mutant, promoted cell migration (**Figure 3I**, top panel). However, knockdown of *SETD3* was able to partially compromise phenotypes of accelerated cell migration caused by overexpression of *PLK1* (**Figures 3I, J**). Meanwhile, cell migration assays also showed similar results, in which overexpression of WT *PLK1*, but not K82M mutant, increased cell migration, whereas knockdown of *SETD3* can rescue the accelerated phenotype caused by *PLK1* overexpression (**Figures 3K, L**). Together, we conclude that SETD3 is required for PLK1-promoted liver cell migration.

### SETD3 Levels Are Positively Correlated With *PLK1* Expression in Human Hepatocellular Carcinoma

**Figure 4:**
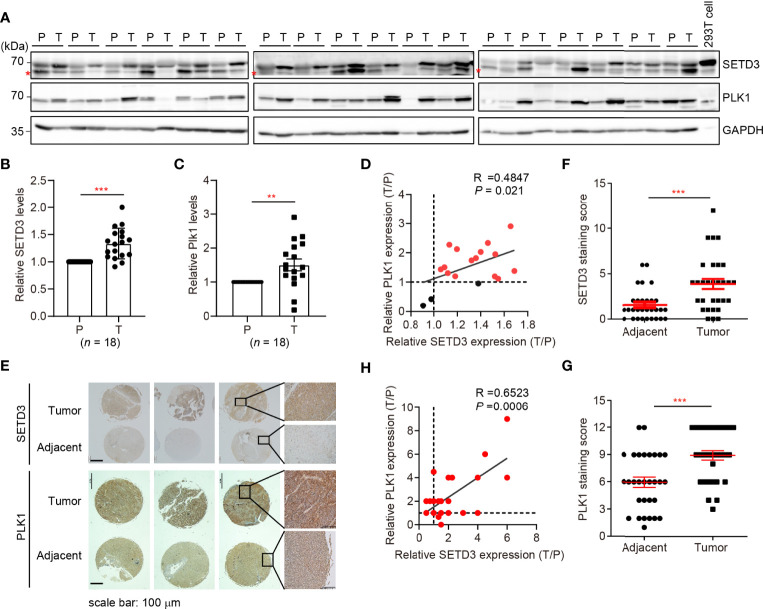
SETD3 levels are positively correlated with PLK1 expression in liver tumors. **(A–C)** Eighteen pairs of human liver samples including adjacent tissues (P) and tumor tissues (T) were examined using Western blotting **(A)**. Relative protein levels of SETD3 **(B)** or PLK1 **(C)** in the tumor tissues compared with the corresponding adjacent tissues were quantified. **(D)** The correlation of SETD3 and PLK1 levels from Western blots in panels **(B, C)** were plotted (R =0.4847, *p* =0.021). The dots represent the relative levels from each tumor sample overlapped as one dot in the graphs. **(E–G)** IHC staining of SETD3 and PLK1 in human liver cancer and adjacent tissues obtained from tissue microarrays (24 pairs). Representative IHC staining images of SETD3 and PLK1 from the same arrays are shown in panel **(E)**. Scale bar, 100 μm. Scores of the staining of SETD3 or PLK1 in panel **(E)** are plotted as in panel **(F)** or **(G)**, respectively. *n* =24. **(H)** The correlation of SETD3 and PLK1 levels from IHC staining in panel **(E)** were plotted (R =0.6523, *p* =0.0006). Data are presented as mean ± SD. **p < 0.01; ***p < 0.001.

Next, we sought to investigate whether upregulated SETD3 and higher PLK1 levels are concurrent *in vivo*. Therefore, 18 pairs of human tumor and adjacent liver tissues were collected, and SETD3 and PLK1 levels were examined by Western blot analysis (**Figure 4A**). We observed that 15 out of 18 pair tissues displayed simultaneously increased SETD3 and PLK1 levels in tumor samples compared to their corresponding adjacent samples (**Figures 4B, C**). Correlation analysis indicated that upregulated SETD3 was positively correlated with higher levels of PLK1 in hepatocellular carcinoma samples (R = 0.4847, *p* = 0.021, **Figure 4D**). To further consolidate this result, commercial purchased liver tumor tissue microarrays containing 24 pairs of human hepatocellular carcinoma and adjacent pairs of tissues were immunostained with α-SETD3 or α-PLK1 antibodies, respectively (**Figure 4E**). Consistently, we observed that higher levels of SETD3 are positively correlated with higher levels of PLK1 (R = 0.6623, *p* = 0.0006, **Figures 4F–H**). Overall, we unveil a positive correlation of SETD3 and PLK1 in human liver tumors.

### Knockdown of Mouse *Setd3* Represses *AKT*-Induced Hepatocarcinogenesis *In Vivo*

Several mouse experimental models have been widely used to simulate hepatocarcinogenesis, including xenograft model, diethylnitrosamine (DEN)-induced HCC model, oncogene-induced HCC model, and transgenic HCC model (30). Compared to DEN-induced or transgenic HCC model that needs over 6 months to induce HCC, an elegant and simple method was developed for liver-specific transgenesis that combines hepatic overexpression of the human oncogene *AKT* and *NRAS* and somatic integration mediated by *Sleeping Beauty* (SB) transposase system, which allowed generation of HCC transgenic model with reduced time and resource (25).

**Figure 5:**
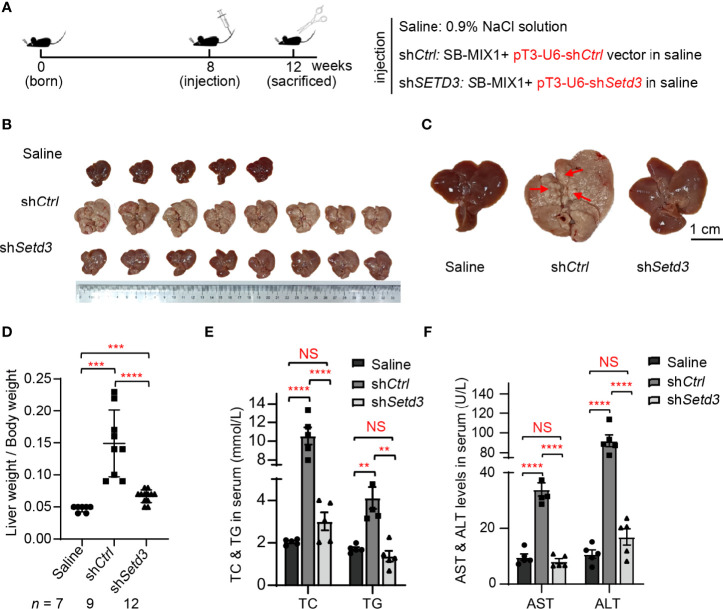
Knockdown of *Setd3* rescued myr-*AKT*- and *NRAS*-induced hepatic tumorigenesis in mice. **(A)** Experimental procedure of development of hepatocarcinogenesis in mice by hydrodynamics-based DNA transfection. Each injection represents delivery of different components as indicated on the right. **(B)** Macroscopic appearance of livers and tumors from mice injected the indicated genes. **(C)** The representative enlarged images of livers from different mice are shown. The red arrows indicate the positions of liver tumors. Scale bar, 1 cm. **(D)** Relative ratios of liver weights vs. body weights in each group of hydrodynamic-injected mice (*n* =7, 9, or 12 in saline, sh*Ctrl*, or sh*Setd3*, respectively). **(E, F)** Quantification of serum TC and TG in panel **(E)** or serum AST and ALT in panel **(F)** of the indicated mice (*n* = 5, per group). Data are presented as mean ± SD. NS, not significant; **p <0.01; ***p <0.001; ****p <0.0001.

Thus, transposons encoding HA-tagged myristylated *AKT* (*myr-AKT*) plus *NRAS-V12* (G12V) were mixed with plasmids expressing the SB transposase (named as SB-MIX1) in the presence of sh*Control* or sh*Setd3* construct and then hydrodynamically delivered to the livers of 8-week-old C57BL/6 male mice *via* tail vein injection. A group of mice was injected with 0.9% NaCl solution as a control. Livers were harvested at 12 weeks post-hydrodynamic injection (**Figure 5A**). As expected, livers in the control mice group looks normal, whereas livers in the mice with *myr-AKT* plus *NRAS* transfected group showed a spotty and paler appearance and were substantially enlarged, suggesting a prominent tumor development (**Figures 5B, C**). However, livers in the mice group injected with *myr-AKT* plus *NRAS* plasmids with sh*Setd3* displayed a rather normal morphology and smaller liver sizes and showed compromised tumor development, although liver sizes were still 1.1-fold larger than the control group, but 3-fold smaller than the sh*Ctrl* group (**Figures 5B–D**). The protein levels of mouse Setd3 in different groups of liver tissues were examined by Western blot, and decreased Setd3 levels were only found in the liver tissue with transfection of sh*Setd3*, but not found in the lung or heart tissue (**Supplementary Figures S1A, B**). Immunochemical staining confirmed that Setd3 levels did not alter in mice transfected with *myr-AKT* plus *NRAS* plasmids and were visually decreased in mice cotransfected with sh*Setd3* and *myr-AKT* plus *NRAS* constructs (**Supplementary Figures S1C, D**). Consistently, the total cholesterol (TC) and total triglyceride (TG) levels in serum were significantly increased in the *myr-AKT* plus *NRAS* group compared to the control group, but decreased upon transfection of sh*Setd3* construct (**Figure 5E**). Moreover, markers of liver injury, such as ALT and AST in serum, also remarkably increased in the *myr-AKT* plus *NRAS* group, but restored to the control levels when co-transfected sh*Setd3* construct (**Figure 5F**). These results indicate that Setd3 is critical for hepatocellular tumor development.

### Knockdown of Mouse *Plk1* Represses *Setd3* Overexpression-Induced Hepatocarcinogenesis *In Vivo*

**Figure 6:**
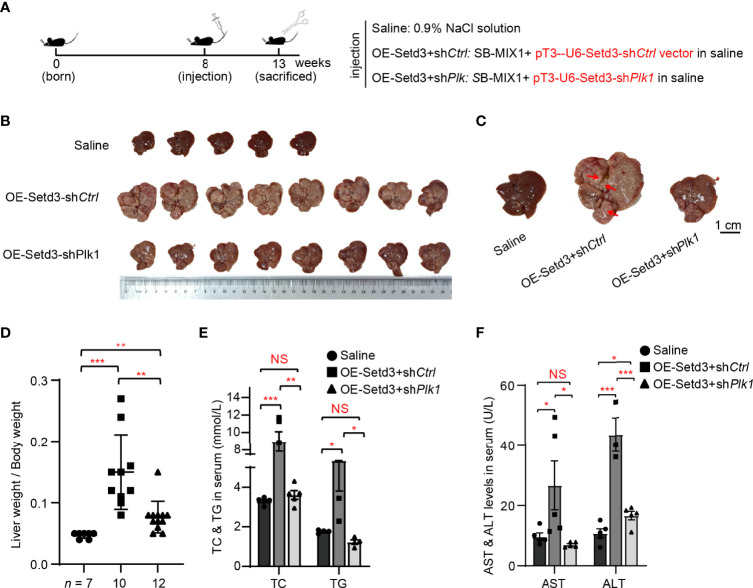
Knockdown of *Plk1* rescued *Setd3* overexpression-induced hepatic tumorigenesis in mice. **(A)** Experimental procedure of development of hepatocarcinogenesis in mice by hydrodynamics-based DNA transfection. Each injection represents delivery of different components as indicated on the right. **(B)** Macroscopic appearance of livers and tumors from mice injected the indicated genes. Scale bar is shown below. **(C)** The representative enlarged images of livers from different mice are shown. The red arrows indicate the positions of liver tumors. Scale bar, 1 cm. **(D)** Relative ratios of liver weights vs. body weights in each group of hydrodynamic-injected mice (*n* =7, 10, and 12 in saline, OE-Setd3+sh*Ctrl*, and OE-Setd3+sh*Plk1*, respectively). **(E, F)** Quantification of serum TC and TG in panel **(E)** or serum AST and ALT in panel **(F)** of the indicated mice (*n* = 5, per group). Data are presented as mean ± SD. NS, not significant; *p <0.05; **p <0.01; ***p <0.001.

To examine whether Plk1 indeed acts at the downstream of Setd3-induced hepatocarcinogenesis *in vivo*, the same hydrodynamics-based injection procedure was utilized (**Figure 6A**). In agreement with the observations shown above, overexpression of *Setd3* in the presence of *myr-AKT* plus *NRAS* constructs facilitated liver tumor development, accompanied with obvious spotty and paler appearance and larger liver sizes, compared to the control group (**Figures 6B–D**). As expected, knockdown of *Plk1* co-transfected with *Setd3* and myr-*AKT* plus *NRAS* constructs was able to restore normal liver morphology and liver sizes (**Figures 6B–D**). The *Plk1* knockdown efficiency and overexpressed *Setd3* levels in different mice groups were validated by Western blot analysis and IHC staining (**Supplementary Figure S2**). We also examined several body indexes that present the liver status in these mice. In line with previous results, serum TC and TG levels and AST and ALT levels were significantly increased in mice with overexpression of *Setd3* relative to the control group, but were restored in mice with sh*Plk1* construct (**Figures 6E, F**). Taken together, we speculate that Plk1 very likely functions at the direct downstream of Setd3 in hepatocarcinogenesis.

### SETD3 Targets *PLK1* Promoter to Regulate *PLK1* Expression

**Figure 7:**
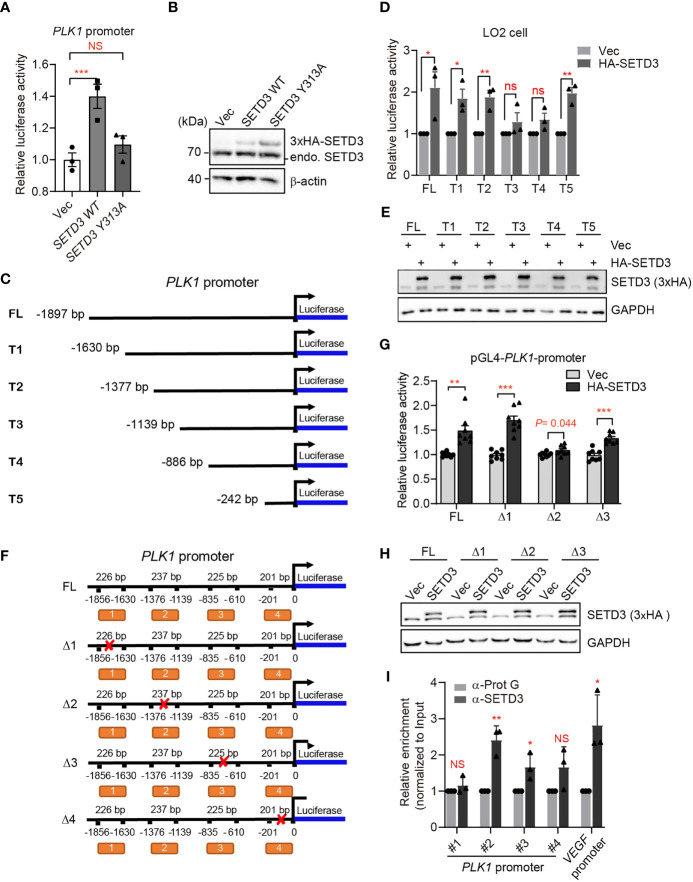
SETD3 targets the promoter of *PLK1* gene to regulate its expression. **(A)** Dual-luciferase assays were performed using the indicated SETD3 constructs, which were co-transfected with a luciferase reporter containing *PLK1* promoter and a Renilla construct. Relative luciferase activity was measured by quantitative PCR. **(B)** SETD3 protein levels expressed in panel **(A)** were examined using Western blotting. **(C, F)** The diagram shows the indicated constructs used in panel **(D)** or **(G)**, respectively. **(D, G)** Dual-luciferase assays were performed using the indicated luciferase reporter constructs bearing different *PLK1*-promoter regions co-transfected with SETD3 and Renilla constructs. **(E, H)** SETD3 protein levels expressed in panel **(D)** or **(G)** were examined using Western blotting, respectively. **(I)** ChIP-qPCR analyses were performed to examine the association of SETD3 with the indicated regions of *PLK1* promoter. The binding of SETD3 with the VEGF promoter served as a positive control. The relative enrichment of SETD3 was normalized to input and relative to the association of protein G beads. Data are presented as mean ± SD. NS, not significant; *p <0.05; **p <0.01; ***p <0.001.

To testify our assumption, we examined whether SETD3 directly regulates *PLK1* transcriptional level using dual-luciferase reporter assays. We engineered the *PLK1* promoter containing ~2,000 bp upstream of *PLK1* coding region into 5′ end of *LUC* gene encoding luciferase as described previously (26) and tested the effect of SETD3 on the activation of *LUC* gene expression. We noticed that the expression of WT SETD3 was able to activate luciferase activity, and the catalytic activity of SETD3 is required for this activation, which does not result from lower protein level of SETD3 Y313A mutant compared with the WT (**Figures 7A, B**). To narrow down the targeted region on *PLK1* promoter by SETD3, we systematically truncated the *PLK1* promoter and generated a series of *LUC* reporter constructs (**Figure 7C**). These reporter constructs were stably expressed in HEK 293T cells, and a 3xHA-SETD3 plasmid was individually transfected into the indicated cells. The relative luciferase activity was examined after 24 h transfection. We noticed that once there is removal of the region of −1,376/−1,139 bp on the *PLK1* promoter, the activation of luciferase expression was impaired (**Supplementary Figure S3**). Intriguingly, if only retaining the nearest 242 bp of the *PLK1* promoter, the luciferase activity was even higher than the longest *LUC* reporter construct (**Supplementary Figure S3**). To rule out a possible heterogeneous effect using different types of cell line, the dual luciferase reporters were co-transfected with 3xHA-SETD3 into immortalized liver LO2 cells. Consistent with previous results, the removal of the region of −1,376/−1,139 bp on the *PLK1* promoter compromised the luciferase activity, whereas the nearest 242 bp of the *PLK1* promoter was also required to activate luciferase gene expression (**Figure 7D**). We confirmed that the epitopic-expressed SETD3 protein levels were relatively comparable in each stable cell line (**Figure 7E**). To validate which region of the *PLK1* promoter is more critical for SETD3 to activate luciferase activity, we constructed several truncated forms of *LUC* reporter gene that lack the indicated DNA fragments (**Figure 7F**), and relative luciferase activity was examined. As shown in **Figure 7G**, deletion of the second region (Δ2, −1,376/−1,139 bp), but not the other two regions (Δ1, −1,856/−1,630 bp; Δ3, −835/−610 bp), is critical for its activation by SETD3. We also confirmed that SETD3 protein levels were comparable in these samples (**Figure 7H**). Intriguingly, deletion of the nearest promoter region of *PLK1* (Δ4, −201/+0 bp) abolished a basal luciferase activity compared to that expressing the empty pGL4 vector alone, suggesting that this region may be essential for binding of certain transcriptional factor. To confirm that SETD3 regulates *PLK1* expression by association of the *PLK1* promoter, ChIP-qPCR analysis was performed. We observed that, compared to the enrichment of SETD3 at the *VEGF* promoter that served as a positive control reported previously, the enrichment of SETD3 at the promoter of *PLK1* gene was also obvious, especially the second but not the fourth region of the promoter, indicating a potential role of SETD3 in *PLK1* expression (**Figure 7I**). Altogether, we conclude that SETD3 could target *PLK1* locus at the −1,400/−1,000 bp region and facilitate its expression.

## Discussion

In this study, we utilized unbiased bioinformatics analysis to find that SETD3 positively regulates the transcriptional levels of many genes, particularly PLK1, in hepatocellular tumor cells (**Figure 1**). We also showed that overexpression of SETD3 promoted tumor cell migration, whereas inhibition of PLK1 activity compromised these phenotypes (**Figures 2**, **3**). Importantly, by taking advantage of a *Sleep Beauty* transposase system, we confirmed that SETD3 levels in the liver are positively correlated with hepatocarcinogenesis *in situ*, but knockdown of *Plk1* attenuates Setd3-caused tumorigenesis in mice model (**Figures 5**, **6**). Mechanistically, we found that SETD3 binds to the promoter of *PLK1* gene and activates its transcriptional level (**Figure 7**). Therefore, our data suggest that elevated SETD3 induces hepatocellular carcinoma by enhancing *PLK1* expression.

PLK1 belongs to a serine/threonine kinase family that plays a critical role in the control of cell cycle progression. Active PLK1 regulates DNA checkpoint, G2/M transition, mitosis, spindle assembly, and cytokinesis. However, overexpressed PLK1 or overactivated PLK1 signaling brings up various defects during cell cycle progression, which leads to multiple tumors, including HCC (31–33). Clinical data indicated that PLK1 levels increased progressively from non-neoplastic liver tissues to HCC, reaching maximal expression in tumors with poorer survival compared with normal livers (34, 35). Considering its crucial functional node in the oncogenic network, PLK1 has been proposed to serve as a potential target for the treatment of cancer. Although several small molecular inhibitor of PLK1 have been used as a chemotherapeutic drug in the clinical trials, less intratumoral efficacy and resistance remain to be omnipresent problems (36, 37). Therefore, other effective druggable targets should be explored further. In this work, we uncover that SETD3 likely regulates PLK1 levels directly, which might provide a combinational way through simultaneous inhibition of PLK1 activity and reduction of PLK1 transcriptional levels to deal with drug resistance of cancer cells.

In recent years, increasing evidence indicates that SETD3 is a histidine methyltransferase but not a lysine methyltransferase, in which only H73 of beta-actin, but not any other residues on other proteins, can be methylated by SETD3 (9). In contrast, a line of studies reported that SETD3 either directly or indirectly regulates expression of multiple genes under different circumstances (11, 14–18). In this work, we found that SETD3 regulates expression of *PLK1* gene (**Figure 1**). We further verified that SETD3 could directly target to the promoter of *PLK1* gene and may facilitate *PLK1* expression (**Figure 7**). Of note, it has been extensively demonstrated that E2F1, FoxM1, p21, p53, or DDX5 directly or indirectly interacts with upstream regions of *PLK1* to modulate its expression, suggestive of dynamic regulation of PLK1 (26, 38–40). Therefore, it is likely that histidine residues on histones or the transcriptional factors could be modified by SETD3, thereby facilitating gene transcription. Indeed, Levy’s group has demonstrated that SETD3 and its activity is required for p53 recruitment to its target genes and for the activation of their expression (14). Therefore, it is likely that SETD3 modulates some unknown transcription factors to regulate *PLK1* expression. More investigation needs to be further explored. It is worthy to point out that lysine methylation commonly served as epigenetic markers to recruit diverse transcriptional modifiers to modulate chromatin structure and subsequently to alter gene expression (41). Currently, whether and how SETD3-mediated histidine methylation regulates gene expression is still unclear. Of note, METLL9 and METLL18, two novel identified histidine methyltransferases, have also been demonstrated to be associated with HCC or gastric cancer (42–44), which implicates a critical role of histidine methylation in cancers.

In this study, we took advantage of SB transposase system with hydrodynamic injection technique to verify the connection of Setd3 and Plk1 upon *in situ* generation of HCC in mice. We clearly showed that overexpression of Setd3 facilitates myr-AKT plus NRAS-dominated hepatocarcinogenesis, whereas knockdown of *Setd3*, at least partly, compromises this phenotypes (**Figure 5**). Since Plk1 is one of the target genes of Setd3, we decided to knock down *Plk1* accompanied with overexpression of Setd3 in the mouse liver to examine the relationship. As expected, the knockdown of *Plk1* alleviated severe liver carcinoma caused by Setd3 (**Figure 6**). Thus, we believe that elevated Setd3-Plk1 axis may be one of the critical signaling pathways involved in HCC. However, whether the enzymatic activity of Setd3 is required for the activation of Plk1 and Plk1-associated HCC should be extensively studied.

In summary, our study provided clear evidence showing that SETD3 upregulation in HCC regulated PLK1 expression, a central player in cell cycle and cell division, which is required for HCC formation and tumorigenesis. We should point out that our study has not fully addressed at what pathological conditions SETD3 is activated to upregulate PLK1 expression and whether the enzymatic activity of SETD3 post-translationally regulates of PLK1 at protein level. In addition, to downregulate *PLK1* expression, the molecular inhibitors of SETD3 should be urgently developed. Recently, actin-based peptidomimetics as an inhibitor of human SETD3 was reported (45). Whether this peptide could be used to effectively treat HCC or whether other small molecule inhibitors of SETD3 can be explored needs further investigation. Nevertheless, given the clinical failure of PLK1 inhibition in the treatment of HCC, the development of small molecular inhibitors of SETD3 might be a promising strategy to inhibit PLK1 expression for the effective treatment of HCC.

## Data Availability Statement

The datasets presented in this study can be found in online repositories. The RNA-seq raw data were deposited in NCBI: https://www.ncbi.nlm.nih.gov/bioproject/PRJNA845942/.

## Ethics Statement

The studies involving human participants were reviewed and approved by the Life and Medicine Ethics Committee of the Wuhan University. The patients/participants provided their written informed consent to participate in this study. The animal study was reviewed and approved by the Animal Experimentations Ethics Committee of the Wuhan University (No. WDSKY0202001).

## Author Contributions

H-ND conceived the project. MC, JB, YL, and M-JZ performed the experiments. QY, JB, and XX collected the patient tissue samples. W-JS and H-ND performed bioinformatics analysis. JS and ZH provided hydrodynamics-based injection method and helped the manipulation. All authors participated in analyzing the data. H-ND, QY, and JB wrote the manuscript. All authors contributed to the article and approved the submitted version.

## Funding

This work was supported by the National Key R&D program of China (2019YFA0802501 to H-ND), National Natural Science Foundation of China (31971231 and 31770843 to H-ND, 31971166 to XX), the Application Fundamental Frontier Foundation of Wuhan (2020020601012225 to H-ND), and the fellowship of China Postdoctoral Science Foundation (2021M702526 to M-JZ).

## Conflict of Interest

The authors declare that the research was conducted in the absence of any commercial or financial relationships that could be construed as a potential conflict of interest.

## Publisher’s Note

All claims expressed in this article are solely those of the authors and do not necessarily represent those of their affiliated organizations, or those of the publisher, the editors and the reviewers. Any product that may be evaluated in this article, or claim that may be made by its manufacturer, is not guaranteed or endorsed by the publisher.
