# Supplementary material for: SETD3 Methyltransferase Regulates PLK1 Expression to Promote In Situ Hepatic Carcinogenesis
Source: Front Oncol. 2022 Jul 14;12:882202. doi: 10.3389/fonc.2022.882202 (PMC9329778; doi:10.3389/fonc.2022.882202)
Supplement: Supplementary file 1 [file DataSheet_1.docx]

Supplementary Material

# Supplementary Tables

**Supplementary Table S1**. Primers used in qPCR

| Primer name | Sequences (5’ to 3’) | purpose |
| --- | --- | --- |
| *SETD3*-F: | GCTTTTGGCTTTTCTCCG | mRNA |
| *SETD3*-R: | AAGTTTGACCTCGTTGTCCC | mRNA |
| *PLK1*-F: | TGACTCAACACGCCTCATCC | mRNA |
| *PLK1*-R: | GCTCGCTCATGTAATTGCGG | mRNA |
| *BOP1*-F: | GAGTATGCGGAGGACAGCTC | mRNA |
| *BOP1*-R: | CGCCAGTAGTCAGGATCGTC | mRNA |
| *PCNA*-F: | CGGCATTAAACGGTTGCAGG | mRNA |
| *PCNA*-R: | TAGCTGGTTTCGGCTTCAGG | mRNA |
| *FOXC1*-F: | CGGGAGATGTTCGAGTCACA | mRNA |
| *FOXC1*-R: | TCCTGCTTTGGGGTTCGATT | mRNA |
| *SF3A2*-F: | TCGACATCAACAAGGACCCG | mRNA |
| *SF3A2*-R: | GCTTCTTICCCCTGCGTATGT | mRNA |
| pGL4-*PLK1* F*:* | CTGAGCTCGCTAGCCTCGAGGCAGCTCCCA  ATGAGAACCCTG | Luciferase assay |
| pGL4-*PLK1* R*:* | ATACCCTCTAGTGTCTAACTCCTCCCCGAATTCAAACGC | Luciferase assay |
| pGL4-*PLK1* ∆1 F: | CCCTGAACACTGAGTCTGTAATGATCATCAGACT  AGGAGGTGGTGGTG | Luciferase assay |
| pGL4-*PLK1* ∆1 R: | CACCACCACCTCCTAGTCTGATGATCATTACAG ACTCAGTGTTCAGGG | Luciferase assay |
| pGL4-PLK1 ∆2 F: | CAAAGTCTTCCTTTTATTACTCAAGTACTACCA  GAACAAACGGG | Luciferase assay |
| pGL4-*PLK1* ∆2 R: | CCCGTTTGTTCTGTAGGTACTTGAGT AATAAAAGGAAGACTTTG | Luciferase assay |
| pGL4-*PLK1* ∆3 F: | ACATGGTGAAACCTCTTCTCTAC TGACCAAGAAACTGAGTGTCCACTA | Luciferase assay |
| pGL4-*PLK1* ∆3 R: | TAGTGGACACTCAGTTT CTTGGTCAGTAGAGAAGAGGTTTCACCATGT | Luciferase assay |
| pGL4-*PLK1* T1 F: | CTGAGCTCGCTAGCCTCGAGtcatcagactaggaggtggt | Luciferase assay |
| pGL4-*PLK1*  T1 R: | CTGAGCTCGCTAGCCTCGAGcagccaacattttagtaagt | Luciferase assay |
| pGL4-*PLK1* T2 F: | acttactaaaatgttggctgCTCGAGGCTAGCGAGCTCAG | Luciferase assay |
| pGL4-*PLK1*  T2 R: | CTGAGCTCGCTAGCCTCGAGactcaagtacctacagaaca | Luciferase assay |
| pGL4-*PLK1* T3 F: | CTGAGCTCGCTAGCCTCGAGactcaagtacctacagaaca | Luciferase assay |
| pGL4-*PLK1*  T3 R: | tgttctgtaggtacttgagtCTCGAGGCTAGCGAGCTCAG | Luciferase assay |
| pGL4-*PLK1* T4 F: | CTGAGCTCGCTAGCCTCGAGtggtgcatgcttgtaattcc | Luciferase assay |
| pGL4-*PLK1*  T4 R: | ggaattacaagcatgcaccaCTCGAGGCTAGCGAGCTCAG | Luciferase assay |
| pGL4-*PLK1* T5 F: | ACCTGAGCTCGCTAGCCTCGAGctctctcggggctgggtctccg | Luciferase assay |
| pGL4-*PLK1*  T5 R: | cggagacccagccccgagagagCTCGAGGCTAGCGAGCTCAGGT | Luciferase assay |
| *PLK1*-promoter-1-F: | GCTTCCCTTGTATACAACATTGCA | ChIP |
| *PLK1*-promoter-1-R: | TGTACTAGGAGATTCACAGACTCA | ChIP |
| *PLK1*-promoter-2-F: | CAGCCAACATTTTAGTAAGTACCC | ChIP |
| *PLK1*-promoter-2-R: | GGCAACCCTGGAACAGTAAAATAC | ChIP |
| *PLK1*-promoter-3-F: | TGGTGCATGCTTGTAATTCCAG | ChIP |
| *PLK1*-promoter-3-R: | TTGGAAGAATGCCTAGCAAACT | ChIP |
| *PLK1*-promoter-4-F: | CTCTCTCGGGGCTGGGTCTCC | ChIP |
| *PLK1*-promoter-4-R: | CTCCTCCCCGAATTCAAACGC | ChIP |
| *VEGF*-promoter-F: | CCCCTTTCCAAAGCCCATTCC | ChIP |
| *VEGF*-promoter-R: | CCTTCTCCCCGCTCCAACACCC | ChIP |

## Supplementary Figures


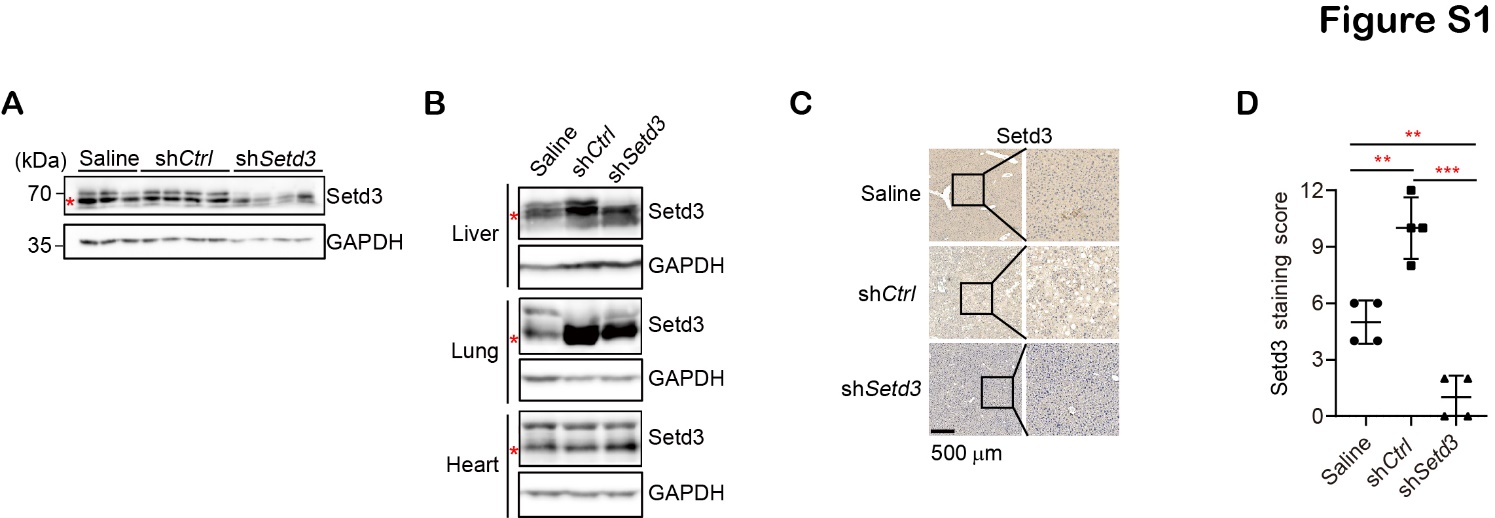


**FIGURE S1** Examine effects by knockdown of *Setd3* in myr-*AKT*- and *NRAS*-induced hepatocarcinogenic mice. (**A**) Western blot analysis to examine knockdown efficiency of *Setd3* in hydrodynamic injected mice. Red asterisk represents non-specific bands. (**B**) Western blot analysis to examine Setd3 levels in different mouse tissues. Red asterisks represent non-specific bands. (**C**) Representative IHC staining images of the liver samples stained with an α-Setd3 antibody from the indicated hydrodynamic injected mice were shown. Hematoxylin was used to co-stain nuclei. The enlarged images were shown on the right. *Scale bar*: 500 μm. (**D**) Scores of the Setd3 staining of mice liver samples of panel **C** (*n* = 4, each group). Data are presented as mean ± SD from four biological samples. ** *P* <0.01; *** *P* < 0.001.


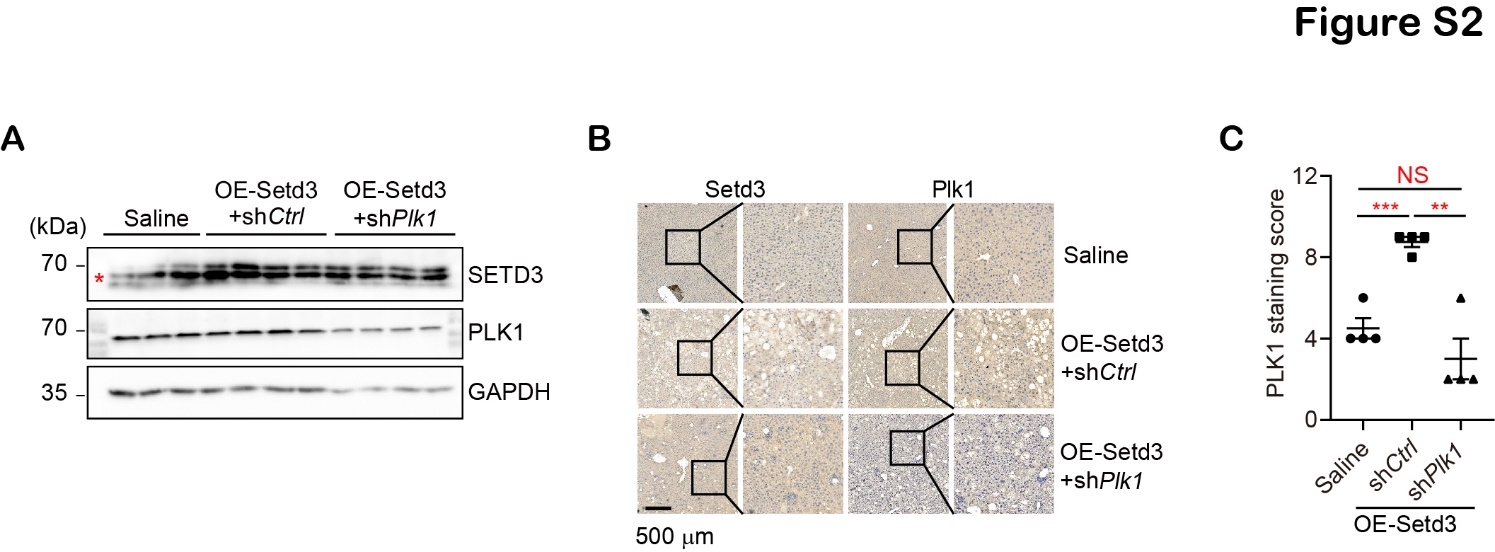


**FIGURE S2** Examine effects by knockdown of *Plk1* in Setd3 overexpression-indcued hepatocarcinogenic mice. (**A**) Western blot analysis to examine Setd3 and Plk1 levels in hydrodynamic injected mice. Red asterisk represents non-specific bands. (**B**) Representative IHC staining images of the liver samples from the indicated hydrodynamic injected mice were stained with α-Setd3 or α-Plk1 antibodies, respectively. Hematoxylin was used to co-stain nuclei. The enlarged images were shown. *Scale bar*: 500 μm. (**C**) Scores of Plk1 staining of mice liver samples of panel **B** (*n* = 4, each group). Data are presented as mean ± SD. NS, not significant; ** *P* <0.01; *** *P* <0.001.

**
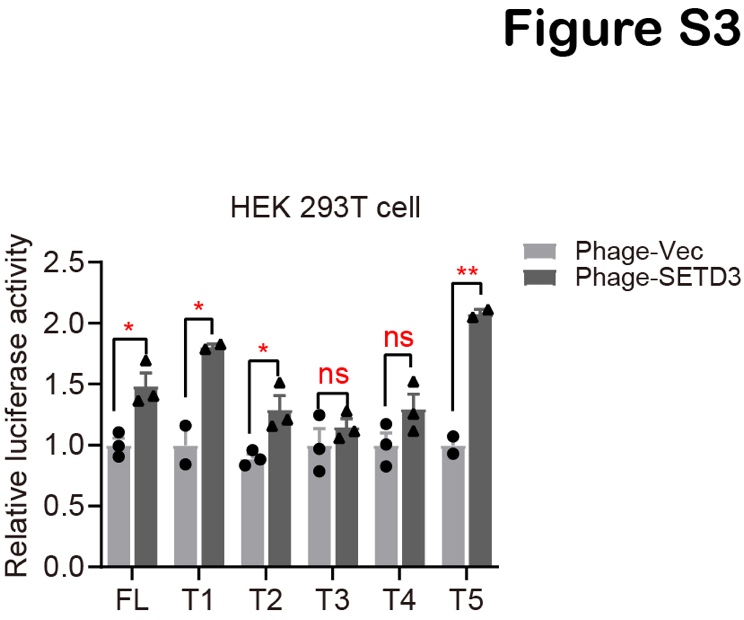
**

**FIGURE S3** Dual-luciferase assays were performed using the indicated luciferase reporters containing individual truncated *PLK1* promoters shown in Figure 7C, which were stably transfected with SETD3 and Renilla constructs in HEK 293T cells. Relative luciferase activity was measured by quantitative PCR.
